# Professional Studies of the Dental Pupil

**Published:** 1857-03

**Authors:** B. P. Aydelott

**Affiliations:** President of the Board of Trustees


### PROFESSIONAL STUDIES OF THE DENTAL PUPIL.

[An address at the Commencement of the Dental College of Ohio, Feb. 25th, 1857. By B. P. AYDELOTT, D. D., President of the Board of Trustees.]

What is meant by Dentistry? It is popularly understood to be the art of pulling and repairing the natural teeth, and of putting in artificial teeth.

But this common view falls far short of the legitimate scope of dentistry, and has doubtless aided much in times past to keep it in public estimate below the position of a profession.

Whence, however, this popular misapprehension? We suppose, it has arisen in part from the name by which the practitioners of this art have hitherto been content to be called; and partly from the fact, that, mere operations upon the teeth are the most obvious and ordinary, though by no means, the sole, or the most important occupation of the dentist.

We would define dentistry to be that part of medical science which treats of disorders of the teeth, and the gums and the mouth generally, and of the means of remedying these. We cannot confine it within narrower limits, because the teeth stand in such intimate and important relations to the other parts of the mouth, that he who does not make himself acquainted with the latter and their diseases, can be but imperfectly qualified to treat those of the former. Whether, for example, a tooth should be extracted or not, or, whether an artificial one ought to be inserted or not, may often depend upon the previous question. What is the state of the maxillary bone, or of the adjacent gum, or of some other of the soft parts of the mouth, or, at times, of the system generally? Now, just as the dentist has mastered these questions, is he prepared for the practice of his calling.

But, if the sphere of the Dentist be so wide, and its relations to medical science generally so intimate and important, it is at once lifted up above the merely mechanic art of pulling and setting teeth, to the position of a profession, a most respectable and responsible profession, one that requires high scientific and literary training for its due cultivation and practice.

In accordance with these views, we took up at our last commencement exercises the inquiry; *What ought to be the preparatory education of the student of dental science?* And we endeavored on that occasion to shew that it should be just that of the other professions, viz: Scientifiic and literary, in a word, the usual college course which has formed nearly all the great minds of the last three or four centuries in medicine, law, divinity, and philosophy generally.

We now propose another question of equal importance. *Ought the student of dentistry to go through a full course of medical science?* We believe that he ought, and the one object of our present discourse is to show this.

Medical science, you are aware, is mainly made up of the following branches. Chemistry, anatomy, physiology, pathology, therapeutics, and materia medica. Under some one or other of these general heads can be arranged all its peculiar studies.

That the dental student ought to give special attention to those subjects about which he is to be immediately engaged in his daily practice, we doubt not; still we maintain that, the study of medical science generally is, not only consistent with this attention, but absolutely necessary to the most intelligent and successful prosecution of his special studies and pursuits.

Our theme then is,—THE DENTAL STUDENT OUGHT TO GO THROUGH A GENERAL COURSE OF MEDICAL SCIENCE. And our first argument is this:—

I. *Dental chemistry* requires it.

Every one at all conversant with the laboratory of the dentist knows, that, to accomplish the processes there going on with intelligence and the highest advantage, demands an acquaintance with the whole range of chemical science. With such knowledge only, can he undertand the *why* and the *wherefore* of all he does; and so will he be best prepared to obviate difficulties, and to introduce better means and methods for obtaining these ends.

But, besides this, a knowledge of the various solids and soft parts which make up the mouth, and of the various secretions poured into it, and of the processes performed in it, and of those of the whole system in which the processes of the mouth are the initiatory step; a knowledge of these, we say, involves the whole subject of animal chemistry. But animal chemistry is so connected with the entire range of chemical science, as to make a careful study of the latter absolutely necessary to a knowledge of the former. Again:

II. *Dental Anatomy* demands an acquaintance with general anatomy.

The human body is made up of a vast variety of parts, external and internal, all admirably adapted to each other, and to subserve the great purpose of the whole system. It is thus, if we may so express ourselves, a *complex unit*, each part of which is dependent upon every other. And hence, the knowledge of one part will greatly facilitate an acquaintance with any other; and the study of the whole will shed additional light upon each individual portion.

Hence, also, it is impossible to be thoroughly acquainted with the structure of any one organ of the human body,—such as the hand, the eye, the mouth, the stomach, the liver,—while ignorant of the anatomy of the whole system. It is for this reason, we suppose that no medical author or professor ever undertook to teach anatomy, by taking up the various members, and giving their structure one after the other. Such a method of instruction would be long, difficult, and obscure; it would necessarily involve repetition at every subsequent step; and at last must prove a miserable failure. The instructor usually begins with osteology, then he proceeds to myology, and splanchnology, and afterwards takes up in order the vascular, the absorbent, and the nervous systems.

How manifest then is it, that he who would become an intelligent and well qualified dentist, must make himself acquainted, not merely with the anatomy of the mouth, but with that of the whole man. Further,

III. In order to a thorough understanding of the *physiology of the mouth*, the dentist must study physiology generally.

Physiology may be defined, the science of life; it treats of the functions of the living system.

But as the system itself is, strictly speaking, a unit, though made up of many parts, so the functions performed by these several parts are all related to each other, and subserve a common end. That end is the sustaining and preservation of life.

The great function of the mouth is mastication, in which is involved not merely the comminution of the food, but the mingling with it of various fluids secreted by the adjacent glands, and poured into that organ in increased quantities during the process of mastication. After this follows digestion or chymification, the function of the stomach; and then chylification; and finally absorption of the chyle by the lactaels opening upon the inner surface of the alimentary canal below the bile and pancreatic ducts. The fluid thus received into the circulatory organs, is by these supplied to all parts of the system, in which it undergoes many changes, fitting it for various uses in the living economy.

But as all these operations are intimately connected together, and mutually dependent upon each other, so we must, in order fully to understand any one function, study it not only by itself, but in its relations to all the other functions, and in its influences too upon the whole system.

Could he then be a competent accoucheur, or a trustworthy oculist, whose physiology extended no further than the eye, or the uterus? No intelligent person would be willing to trust himself in the hands of such a practitioner, if he knew how limited and imperfect was his professional information. For the same reason the dentist also should embrace not merely the mouth, but the whole system in his physiological studies. Another argument:

IV. *Dental Pathology* cannot be thoroughly understood, without an acquaintance with general pathology.

Pathology treats of various diseases to which the human system is liable, their symptoms, their causes, both predisposing and exciting, their courses, and their issues. In a word, as physiology introduces us to a knowledge of the functions in their healthy state, so pathology makes us acquainted with these same functions in a disordered state. It might, therefore, be reasonably expected, that as the physiology of one part stands so closely connected with the physiology of the whole, that each sheds light upon the other, and consequently the study of both is necessary to the perfect understanding of either; so also the various parts of pathology must sustain intimate relations to each other, and must all be faithfully studied if we would acquire a thorough knowledge of any one.

But in fact, my young friends, this remark is more obviously true when applied to pathology; because the relations existing between various and often distant parts of the system, are generally far more apparent in disease than in health. Hence the importance of the general study becomes here more manifest.

For example, little or no connection is usually perceived in health between the brain and the stomach, so that the physiology of the two may seem to be very little related; but let the stomach become disordered, and how quickly will the brain sympathize with it! Hence not merely medical men, but almost every one knows that the real seat of that most distressing malady, the sick head-ache, is almost always the stomach.

But are not the foregoing remarks emphatically true of the mouth? This part is so closely connected with the whole system, that its functions cannot be disordered without speedily involving other organs, and sooner or later, affecting the whole system. Neither can any general disease exist, nor the action of any other organ or part be long disturbed, without shewing itself in the mouth. Hence the intelligent physician will rarely make up his judgment upon a disease, or be willing to prescribe for it, without first examining the state of the mouth. He finds there some of the most significant and reliable criteria of disease. The mouth itself may indeed be the true seat of the disorder; or, what is most commonly the case, though the mouth may not have been primarily diseased at all, yet it will quickly sympathize with the disordered part however remote, or soon become involved in some general malady, such as a fever, affecting the entire system.

But if these things be so, it follows that the dentist, as well as the physician, ought faithfully to make himself acquainted with the pathology of the whole system, if he would be adequately prepared for his peculiar and important duties. Again:

V. The *therapeutics of the dentist* must extend to the whole system.

The truth of this remark in its application to other branches of the profession is now almost universally acknowledged. The intelligent surgeon, for example, knows that he would be poorly qualified for his duties, if his therapeutics embraced nothing more than those lesions of different parts which he is usually called to treat. He must be versed in therapeutics generally, to enable him with the highest advantage to come to the practice of his profession.

And even where the surgeon limits himself to the treatment of the maladies of a single organ, as in the case of the oculist, or the aurist, he is aware that he cannot be safely ignorant of general therapeutics.

The reason of this, my young friends, is very clear: all parts of the system being as we have seen, more or less intimately connected anatomically, physiologically, and pathologically, it follows that a local therapeutic can never be other than a very imperfect and unsafe guide. He cannot be fully competent to treat any local affection, who knows nothing of the general principles of curative science. A general therapeutics will always shed additional light upon the therapeutics of each particular part.

About forty-two years ago, when connected with the New York city dispensary, I was called upon at that institution by a very respectable lady, to prescribe for her little daughter. The child was about five years old, and had been for a considerable time troubled with a severe ophthalmic affection. The family physician, a reputable practitioner, had used the ordinary local remedies in vain.

After carefully inquiring into the case it struck me, that had the disease been really in the eye, or its appendages, it must have yielded to the treatment employed. Certainly, nothing more of that kind could be done. Some other plan of treatment must be had recourse to, or the child be given up to permanent suffering, and perhaps to ultimate and entire blindness. It occurred to me that the local disorder was not at all *idiopathic*, but merely *symptomatic*; that the alimentary canal was really the seat of disease and vermes the exciting cause. Accordingly I used nothing for the eyes, but prescribed the ordinary anthelmintic remedies. In a short time every symptom disappeared, and the patient was entirely relieved from a distressing malady,

We need here only add, that opthalmia, otitis, and many other maladies, still more remote from the mouth, have often their true origin and seat in a carious tooth, or a diseased maxillary bone. We are sure, therefore, that the experience of every intelligent dentist, who has been but a few years engaged in the practice of his profession, will supply him with many instances demonstrating the value of his general therapeutics. He felt in such cases, that he could not safely and creditably get along without such knowledge. Once more:—

VI. The *materia medica* of the dentist requires an acquaintance with the whole science of materia medica.

There is already a considerable range of preventive and remedial agents employed by the dental practitioner, and this range is continually increasing.

But what he does employ, even in the present state of the profession, is so connected with other articles of the pharmacopœia, that a knowledge of the whole is becoming more and more necessary, to enable him intelligently and efficiently to use those which his daily practice now calls for.

But there are other reasons why such general knowledge is needful to him. Cases, which sometimes fall under his care, either are or have been the subjects of medical treatment by the physician for some general disorder of the whole system, or some local disease. Without then, a certain degree of acquaintance with the properties and influence of the remedies previously employed, or at that time used, the dentist would frequently be at a loss to know what he himself ought to do; and especially is such knowledge indispensable to him, where he is brought into conference with the physician in respect to the condition and treatment of the patient. In order that his opinions may deserve consideration, and he be enabled to sustain himself respectably as a professional man, he ought in such cases to be competent, not only to manage his own particular department, but to form enlightened views of the whole case, or, at least, to comprehend those of the physician with whom he is associated. In this way only, can they harmoniously coöperate, and so give the patient the best chance of recovery.

But there is also a general view of this subject, which ought not here to be overlooked. Materia medica, like every other branch of human knowledge, is continually growing. The medical properties of bodies, are constantly coming to light. And in this respect scientific men, it ought to be acknowledged, owe much to popular observation and popular experience. These are usually ahead of books. And it is the business of the enlightened medical practitioner, whether he be a dentist, a surgeon, an oculist, an accoucheur, or a physician; it is his business, we say, to examine into these popular opinions and test the character of popular remedies, and see in what cases these are worthy to be adopted by the profession.

The *vaccine virus*, for example, as a preventive of small-pox, was long known by the common people in a dairy district of England. It was this traditional belief of the people that excited the notice of Dr. Jenner. After a thorough examination he was convinced of its truth, and called the attention of the profession to it. Who can appreciate the blessings which this discovery in materia medica has conferred upon the human family!

Take another instance. More than a half a century since, the people of a farming district in Pennsylvania were acquainted with the fact, that *creosote* would in many cases remove the toothache. The article had not yet been adopted into the dispensatory; indeed, chemistry had not yet discovered it; but the common people used it successfully, how long indeed before the time I have mentioned, I know not.

The popular directions in respect to the preparation of the remedy and the mode of using it, were as follows: Take a small, green hickory switch; set one end of it on fire, and when a coal has been formed upon it blow out the flame, and hold over the smoking end the inverted bowl of a spoon. This will soon be covered with drops of a straw colored fluid, or, as it was termed, "*sweat*," which is to be carefully wiped off with a small piece of cotton, and inserted into the affected tooth.

We now know that the medical virtues of this rudely obtained liquid resided in the *creosote*, which scientific chemistry has since presented to us in its separate and pure state, and which is thus commonly used in medical practice.*

Let the dentist then, we say, make himself at home in the whole range of the materia medica; and not content with this, let him extend his knowledge as far and as fast as he can by his own observation, and by carefully treasuring up every well ascertained truth which the popular intelligence supplies. There is no danger of his knowing too much here; his materia medica cannot be too wide. But we have done with our argument.

A very important question now arises—it is this: *Where may the dental pupil best prosecute the professional studies we have pointed out?*

Some have thought it most advisable to attend at least one full course of lectures in a regular medical college, and then go through the ordinary course prescribed in a dental college, for the larger acquisition of those particular branches which his department of the profession requires.

Certainly in the state of things till lately existing, such a course was highly expedient, but it may well be doubted whether the day has not come when the dental college may

* Since penning the above paragraph, Dr. Mighels of this city, informed me that the common people of the State of Maine, upwards of half a century since, used the same remedy for odontalgia. They, however, obtained it in the shape of *soot* scraped from the bottoms of pots and kettles.

do all, or nearly all, for its pupils that the public expect from them.

We feel quite confident that in this college the student may find everything he needs, even now, to qualify himself in the highest degree, for his professional duties, except that more extended instruction which the Professor of the Theory and Practice of Medicine, and the Professor of Surgery are required in their several chairs to communicate, in a regular Medical College.

We believe therefore that he needs the advantages of no other institution, except in the two branches just indicated, viz: the Theory and Practice of Medicine, and Surgery. Three Professors here—one of Dental Chemistry and Materia Medica, one of Dental Anatomy and Physiology, and one of the Principles and Practice of Dentistry. These will supply him with everything peculiarly professional. It needs scarcely be added that the first two chairs, while going into the whole subject of their several sciences, should each give his teaching a special bearing and a particular fullness upon all that peculiarly belongs to the knowledge and practice of Dentistry.*

You will readily perceive, my young friends, that we have lifted up a high professional standard. The objects which you are required to keep in view, and the scientific acquirements necessary for the attainment of these, will demand long and arduous study. Indeed your whole life should be one of study, if, in this day of progress, you would keep up with the advance of knowledge, and thus sustain your own respectability, and reflect honor upon your profession.

What then can bring up the mind, the heart—the whole man—to such a work, and keep him steadfast in its prosecution?

---

* The above scheme of Dental Professorship is not given as the only one, or even as the best under all circumstances, that could be devised; but it is presented as a generally desirable and appropriate arrangement. At least the *subjects* embraced in it must be included in the studies of every well organized Dental College.

Some place great reliance upon a love of knowledge either natural, or inspired by judicious tutoring. But while we cheerfully acknowledge the value of this in its place, and would always give it due encouragement, yet, let it not be forgotten that, few have it in great strength naturally, and it is not always to be imparted by any training, to such an extent as so arduous a study demands. And let it be remembered also, that a mere thirst for knowledge does not necessarily lead to a judicious and faithful application of it. A man may be exceedingly learned in his profession, and yet a very indifferent practitioner.

Others have relied mainly upon ambition or the desire of distinction. This also, we believe, has its lawful place. It is not wrong to wish to be held in honor;—indeed it would argue a want of one element of the true nobility of human nature to be insensible to the worth of well merited commendation.

But has not all experience proved that, in the great majority of individuals this principle is not to be relied upon? Hence so very few even of the most highly favored, do ever in this world's struggle really attain to distinction.

Moreover, ambition—to say nothing of the perilous influence it is sure to exercise upon fallen creatures such as we are—ambition rarely exists in such a degree as to make it a reliable ground upon which the teacher may build his hopes of success for his pupils.

We know of only one all-sufficient source of principles to guide, and of motives to stimulate the student and the professional man, in the laborious and self-denying path before them. It is *christianity*, *christianity*—we mean the CHRISTIANITY OF THE BIBLE.

Hence this Institution, while careful to give as full and thorough a course of professional instruction as possible, and to hold out every proper and honorable inducement to exertion, which human nature supplies, has always, as her last authoritative act of kindly solicitude, placed in the hands of each of her pupils, besides his well-earned diploma, a copy of the Holy Scriptures.

In officially carrying out this benign rule on the present occasion, permit, my young friends, one who stood forty-two years ago in a medical college just in the position which you occupy here this evening, and who has since passed through a life of diversified, and often painful experience, and one who has sustained most important and responsible relations to the students of this College ever since its organization, permit such an one at this momentous crisis of your life, solemnly and affectionately to urge upon you the daily and faithful study of the Holy Scriptures. As dying but immortal, as sinful but redeemed creatures, all hastening forward to a judgment seat and the never-ending issues thereof—do we not need such study? In the Holy Scriptures alone are life and immortality brought to light. In them alone have we the great charter of our salvation in *this life*, as well as in that to come, through the atoning blood and renewing spirit of our Lord Jesus Christ. And hence in the Holy Scriptures alone have we a perfect law to guide us, and in them alone are we furnished with motives all sufficient to excite and sustain us in every path of duty.

